# 非小细胞肺癌术后辅助化疗研究进展

**DOI:** 10.3779/j.issn.1009-3419.2014.06.12

**Published:** 2014-06-20

**Authors:** 娜 韩, 红阳 卢

**Affiliations:** 310022 杭州，浙江省肿瘤医院，浙江省胸部肿瘤（肺、食管）诊治技术研究重点实验室 Zhejiang Cancer Hospital, Zhejiang Key Laboratory of Diagnosis and Treatment Technology on Thoracic Oncology (Lung and Esophagus), Hangzhou 310022, China

**Keywords:** 肺肿瘤, 辅助治疗, 化学治疗, Lung neoplasms, Adjuvant therapy, Chemotherapy

## Abstract

肺癌可分为非小细胞肺癌（non-small cell lung cancer, NSCLC）和小细胞肺癌，其发病率和死亡率均高居恶性肿瘤首位。本文主要就NSCLC术后辅助化疗的地位确立、辅助化疗的合适人群和化疗方案选择以及相关生物标志物研究进展作一综述。

## Abstract (en)

Lung cancer including non-small cell lung cancer (NSCLC) and SCLC is the most commonly diagnosed cancer and leading cause of cancer-related death worldwide. This review focuses on progress of the effect, indications, regimens and the related biological markers of postoperative adjuvant chemotherapy in NSCLC.

肺癌发病率和死亡率均高居全球恶性肿瘤的首位^[1]^，可分为非小细胞肺癌（non-small cell lung cancer, NSCLC）和小细胞肺癌，NSCLC的比例呈上升趋势^[2]^。手术是早、中期NSCLC的主要治疗手段，术后辅助化疗有助于提高生存期，改善预后。本文主要就NSCLC术后辅助化疗研究进展作一综述。

## 1 辅助化疗地位确立、适应证及方案

Ⅰ期-Ⅲa期NSCLC术后仍然会出现局部复发或远处转移，人们尝试使用辅助化疗来减少复发或转移，改善预后。1995年非小细胞肺癌协作组（Non-small Cell Lung Cancer Collaborative Group, NSCLCCG）进行了一项包括52项临床研究的荟萃分析^[3]^，结果显示应用烷化剂术后辅助化疗对生存无获益，相对死亡危险比增加15%，5年生存率降低5%；术后行含铂方案辅助化疗较单独手术相比，2年、5年绝对获益率分别为3%和5%（*P*=0.08），相对死亡风险减少13%。ALPI（Adjuvant Lung Project Italy Trial）临床试验入组Ⅰ期-Ⅲa期NSCLC术后患者1, 209例，术后随机分组行丝裂霉素、长春地辛和顺铂联合化疗或临床观察，结果显示术后辅助化疗未能提高总生存期（overall survival, OS）^[4]^。随后在英国进行的BLT试验也得到了阴性的结果^[5]^。该两项研究都未能证实辅助化疗的优势。IALT试验^[6, 7]^共入组1, 867例Ⅰ期-Ⅲ期NSCLC术后患者，随机分为以顺铂为基础的联合方案辅助化疗组或临床观察组，结果显示术后化疗组对比观察组改善了5年生存率，5年无病生存期（disease-free survival, DFS）也有优势（39.4% *vs* 34.3%, HR=0.83, 95%CI: 0.74-0.94, *P* < 0.003），中位随访7.5年的随诊结果显示辅助化疗组的生存优势不明显，DFS则显示出了持续的获益。本试验的结果提示术后辅助化疗能够改善5年的生存情况，而5年以后对生存的延长作用越来越小。该研究为NSCLC术后辅助化疗的发展奠定了基础。IALT研究是在NSCLC术后辅助化疗中规模最大的大样本随机对照试验，并进一步确立了NCSLC术后辅助化疗的地位。

上述研究基本确立了NSCLC术后辅助化疗的地位和作用，术后辅助化疗能够使患者生存获益逐步得到公认，不同分期和不同药物之间获益是否有差别也逐渐成为关注的焦点。JBR-10试验^[8]^共入组Ⅰb期（T2N0）或Ⅱ期（T1N1或T2N1）NSCLC术后患者482例，随机分为长春瑞滨联合顺铂（NP）术后辅助化疗组和观察组。中位随访9.3年的数据显示化疗组生存获益（HR=0.78, 95%CI: 0.61-0.99, *P*=0.04），亚组分析显示这种获益局限于有淋巴结转移（N1）患者，Ⅰb期肿瘤直径≥4 cm与获益相关（*P*=0.022），直径 < 4 cm者不获益（HR=1.73, *P*=0.06），辅助化疗明显降低肺癌死亡风险（HR=0.73, 95%CI: 0.55-0.97, *P*=0.03）。该试验是第一个对所有患者均采用第三代化疗药物的临床试验。与JBR-10相似的ANITA（Adjuvant Navelbine International Trialist Association）临床试验^[9]^得到了相似的结果。美国癌症和白血病B组（Cancer and Leukemia Group B）的CALGB9633临床试验^[10]^研究Ⅰb期NSCLC术后辅助化疗能否获益，共入组344例Ⅰb期患者，随机分为紫杉醇联合卡铂化疗组和观察组，中位随访34个月，化疗组的4年生存率改善达12%，较对照组明显延长（*P*=0.028）；中位随访74个月，化疗组和观察组的5年生存率分别为60%和58%（HR=0.83, 90%CI: 0.64-1.08, *P*=0.125），探索性分析表明肿瘤直径≥4 cm患者辅助化疗获益（HR=0.69, 90%CI: 0.48-0.99, *P*=0.043）。关于NSCLC术后辅助化疗的LACE（Lung Adjuvant Cisplatin Evaluation）荟萃分析^[11]^显示Ⅰa期不获益，Ⅰb期获益不明显，Ⅱ期和Ⅲ期获益明显；不同年龄层（< 69岁、65岁-69岁、 > 70岁）和不同化疗药物之间（包括长春碱类、VP-16）生存获益无统计学差异。这些研究进一步验证了以铂类为基础的辅助化疗可以延长患者OS和DFS。AJCC第7版肺癌TNM分期系统中T2bN0M0（肿瘤直径 > 5 cm，≤7 cm）患者分期由原来的Ⅰb期改为Ⅱa期，CALGB9633临床试验中提到的肿瘤直径≥4 cm的Ⅰb期患者在新的分期中多数成了Ⅱa期。美国国立综合癌症网络（National Comprehensive Cancer Network, NCCN）指南2014年第2版推荐对于有高危险因素的T2abN0患者可行术后辅助化疗（2B推荐），高危因素包括分化差（包括分化好的神经内分泌癌）、脉管浸润、锲形切除、肿瘤直径≥4 cm，脏层胸膜侵犯及淋巴结受累等。

Chu等^[12]^报道的一项NSCLC术后随机分为NP或多西他赛联合顺铂（DP）化疗的Ⅱ期临床试验初步结果显示，中位随访20个月，DP组和NP组的临床疗效和安全性相当。TREAT研究^[13]^是一项关于培美曲塞联合顺铂与NP对照辅助治疗早期NSCLC的Ⅱ临床试验，主要终点指标为临床可行性，次要终点指标为化疗完成情况和安全性等，结果显示培美曲塞联合顺铂安全可行，比NP方案毒性小，化疗完成情况好，但未做化疗疗效比较。Uramoto等^[14]^进行了紫杉醇联合卡铂与吉西他滨联合卡铂随机对照辅助治疗完全切除NSCLC的Ⅱ期临床试验，主要终点指标为方案的依从性，次要终点指标为安全性和毒性。共75例患者入组，该研究认为紫杉醇联合卡铂或吉西他滨联合卡铂双周方案依从性好，安全可行，毒性可耐受。上述研究多为Ⅱ期临床试验，且主要观察方案的依从性，疗效未作为其主要终点指标，有待Ⅲ期随机对照临床试验验证。培美曲塞、紫杉醇、多西他赛等第三代化疗药物联合顺铂与NP方案一样也被美国NCCN和美国临床肿瘤学会（American Society of Clinical Oncology, ASCO）推荐可用于NSCLC术后辅助化疗。

优福定（UFT）是日本研制的一种口服化疗药物，由替加氟和尿嘧啶按1:4的比例混合制成，通过阻断DNA合成来抑制癌细胞生长，副作用较小。日本学者设计的WJSG Ⅱ（West Japan Study Group for Lung Cancer Surgery Ⅱ）临床试验^[15]^入组323例Ⅰ期-Ⅲ期术后NSCLC患者，随机分组进行UFT单药、长春地辛联合顺铂化疗后序贯UFT或临床观察，显示术后UFT单药辅助化疗生存最佳。同样另外一项有关UFT的临床研究（The Japan Lung Cancer Reserch Group Study, JLCRG）^[16]^显示术后Ⅰ期肺腺癌患者UFT口服2年对比临床观察，5年总生存分别为88%和85%（HR=0.71, 95%CI: 0.52-0.98, *P*=0.04），T2患者中这种优势更明显（*P*=0.005）。UFT用于NSCLC术后辅助化疗在欧美国家的认可度相对较低，NCCN指南中也未推荐，国内也鲜有学者在术后辅助化疗中使用UFT，尚需更多的临床试验证据通过与NP/GP/DP等指南中推荐的化疗方案比较来进一步验证UFT的疗效。

Wisnivesky等^[17]^通过研究3, 324例年龄 > 65岁Ⅱ期-Ⅲa期老年肺癌患者进行术后辅以铂类（*n*=684）或无铂类药物为基础的辅助化疗的生存期和不良反应发生率，发现不论放疗与否，化疗可提高生存期（危险比：0.78-0.81），但对于年龄≥80岁者无益，辅助化疗使严重不良反应事件发生率增加2倍。Gu等^[18]^通过对SEER数据库16, 420例年龄 > 65岁老年Ⅰb期-Ⅲa期NSCLC分析，显示11%的患者接受含铂方案辅助化疗，其中83%的患者接受含卡铂方案化疗，含铂方案化疗患者比未行化疗患者明显提高生存期，含卡铂化疗患者与含顺铂化疗患者OS相当，含卡铂化疗患者脱水与贫血的发生率低。然而，由于目前老年人在入组辅助化疗的临床研究中比例少于10%，同时考虑到并发症、耐受性等问题，老年人的辅助化疗中以卡铂替代顺铂的方案值得进一步的研究。

## 2 生物标志物

切除修复交叉互补基因1（excision repair cross-complementation group1, ERCC1）位于染色体19q13.2-13.3，其表达产物与DNA修复酶缺乏互补基因F（XPF）形成紧密的异二聚体（ERCC1-XPF），具有损伤识别和切除5c端的双重作用，在核苷酸切除修复中起到限速或调节的重要作用。核糖核苷酸还原酶M1（ribonucleotide reductase, *RRM1*）基因定位于染色体11q15.5区域，是DNA合成通路中的限速酶。Bepler等^[19]^对来自IALT试验的784例患者术后肿瘤组织ERCC1和RRM1进行自动定量分析检测，其中Ⅰ期270例，Ⅱ期180例，Ⅲ期334例，术后观察组382例，术后足叶乙甙联合顺铂辅助化疗218例，NP化疗122例，长春地辛联合顺铂25例，长春花碱联合顺铂37例。763例患者进行了ERCC1检测，观察组中ERCC1高表达患者与低表达患者生存期无统计学差异（HR=0.77, *P*=0.10, OS; HR=0.80, *P*=0.12, DFS），辅助化疗组ERCC1低表达患者从辅助化疗受益（HR=0.73, *P*=0.02, OS; HR=0.76, *P*=0.04, DFS）。752例患者进行了RRM1检测，RRM1表达水平对OS和DFS无影响，辅助化疗组中RRM1低表达患者有延长生存的趋势（HR=0.84, *P*=0.25, OS; HR=0.87, *P*=0.32, DFS）。ERCC1和RRM1均高表达患者188例，ERCC1高表达和RRM1低表达患者138例，ERCC1低表达和RRM1高表达患者169例，ERCC1和RRM1均低表达患者235例，25例患者ERCC1或RRM1结果丢失；观察组中根据上述ERCC1和RRM1表达情况分的4个亚组在OS上无差异；辅助化疗组中，ERCC1和RRM1均低表达患者生存期最好，但是仍然未达到统计学差异（*P*=0.08）。一项关于ERCC1表达水平对NSCLC患者预后及预测判断作用的*meta*分析^[20]^显示术后未行辅助化疗患者中ERCC1高表达患者OS长于低表达患者（HR=0.69; 95%CI: 0.58-0.83; *P* < 0.001），术后曾行辅助化疗患者ERCC1高表达与低表达患者之间OS无差异（HR=1.41; 95%CI: 0.93-2.12; *P*=0.106）。但是Friboulet等^[21]^利用8F1抗体检测一组494例患者的验证样本，并对已得出了ERCC1表达缺失与铂治疗反应初始相关结论的589例患者的全部原始样本进行复染并与之前的研究结果进行了比较，同时研究者还通过16种ERCC1抗体对其表位进行定位。研究结果显示使用现有ERCC1抗体未能特异性检出具有独特功能性的ERCC1同型异构体，不能证实ERCC1蛋白免疫组化染色有预测效应。这一结果提示抗体的特性有所改变，因此，这种方法对指导治疗决策的作用具有一定的局限性。在2013年ASCO会议上有研究^[22]^指出，在NSCLC术后辅助化疗中，由于缺乏化疗靶向性和严重的不良反应，ERCC1表达并不能预测患者术后辅助化疗的获益。目前临床上不推荐根据ERCC1的表达来判断预后和预测顺铂的疗效。

p27是细胞周期依赖性激酶抑制剂家族成员之一，IALT中发现p27阴性患者接受术后辅助化疗较术后观察能延长生存期（HR=0.66; 95%CI: 0.50-0.88; *P*=0.006），p27阳性患者未能从术后辅助化疗中获益（HR=1.09; 95%CI: 0.82-1.45; *P*=0.54）^[23]^。MutS同源蛋白2（MutS homologue 2, *MSH2*）为错配修复基因，位于染色体2p16-22，其基因组DNA全长约73 kb，在新链DNA合成碱基配对的错配中起修复作用。IALT试验中发现MSH2低表达患者接受术后辅助化疗相比观察能延长生存期（HR=0.76; 95%CI: 0.59-0.97; *P*=0.03），MSH2高表达患者接受辅助化疗相比术后观察也有延长生存期的趋势（HR=1.12; 95%CI: 0.81-1.55; *P*=0.48）^[24]^。在JBR-10临床试验中^[25]^，482例患者中的253例Ⅰb期-Ⅱb期NSCLC采用免疫组化法行抑癌基因*p53*表达水平检测，与单纯手术相比p53表达患者能从辅助化疗中获益（HR=0.54; 95%CI: 0.32-0.92; *P*=0.02），p53不表达患者未能从辅助化疗中获益（HR=1.40; 95%CI: 0.78-2.52; *P*=0.26）；*p53*突变检测发现397例患者中124例（31%）患者有突变，但对辅助化疗无预测价值；另外该试验对450例患者进行了*KRAS*突变检测，发现24%患者存在突变，*KRAS*野生型患者辅助化疗相比观察能延长OS（HR=0.69; 95%CI: 0.49-0.98; *P*=0.03），*KRAS*突变患者辅助化疗未能延长OS（HR=0.95; 95%CI: 0.53-1.71; *P*=0.87）。在今后NSCLC术后辅助化疗临床试验中可根据生物标志物筛选患者行前瞻性研究，并进一步明确相应分子标志物的作用，更好的做到个体化辅助化疗^[26]^。

众所周知，*EGFR*基因突变为个体化EGFR-TKI靶向治疗提供了全新思路。Li等^[22]^进行了培美曲塞和卡铂化疗联合吉非替尼与单用化疗随机对照术后辅助治疗具有*EGFR*突变的Ⅲa-N2 NSCLC Ⅱ期临床试验研究，各30例患者，所有患者均有EGFR外显子19缺失或L858R点突变，结果显示联合组比单用化疗组明显延长PFS（39.8个月*vs* 27.0个月，*P*=0.014）；两组OS无差异，联合组的主要不良反应为皮症（43.3%）；2年的PFS和OS联合组为78.9%和92.4%，单用化疗组为54.2%和77.4%。RADIANT研究拟入组1, 150例Ⅰb期-Ⅲa期完全切除无放疗的NSCLC，多有患者经FISH和/或免疫组化证实EGFR阳性，在术后常规辅助化疗基础上比较厄洛替尼与安慰剂的有效性，研究正在进行中。

## 3 小结

以上众多临床试验（表 1）结果显示NSCLC术后辅助化疗的疗效确切，主要适用Ⅱ期-Ⅲa期患者，对于Ⅰa期患者目前不推荐化疗，Ⅰb患者化疗应慎重，肿瘤直径≥4 cm是一重要考虑因素，化疗方案为NP或其他第三代新药联合顺铂，对于相同病理类型患者采用不同化疗方案获益是否有差异，老年患者也可以从含铂方案辅助化疗中获益，但是需权衡化疗毒性与延长生存之间的利弊关系。其他分子标志物，如ERCC1、RRM1、β微管蛋白及EGFR等对于化疗药物选择的作用等均有待进一步明确，目前基于分子标志物设计的一些临床试验也正在进行中（表 2）。

**1:** Randomized trials and meta-analyses of adjuvant chemotherapy for NSCLC

Trials and *meta* analyses	No. of patients	Phase of clinical trial	Stage	Agents	Hazard ratio (95%CI)	*P*	Advantage of 5-year survival
1995 *meta*-analysis^[3]^	1, 394	*Meta*-analysis	Ⅰ-Ⅲa	CDDP based	0.87 (0.74-1.02)	NS	5
ALPI^[4]^	1, 209	Ⅲ	Ⅰ-Ⅲa	MVP	0.96 (0.81-1.13)	NS	1
BLT^[5]^	481	Ⅲ	Ⅰ-Ⅲa	CDDP based	1.02 (0.77-1.35)	NS	-
IALT^[6]^	1, 867	Ⅲ	Ⅰ-Ⅲ	CDDP+Vin	0.86 (0.76-0.98)	< 0.03	4.1
JBR. 10^[8]^	482	Ⅲ	Ⅰb-Ⅱ	CDDP+VNB	0.69 (0.52-0.91)	0.04	15
ANITA^[9]^	840	Ⅲ	Ⅰb-Ⅲa	CDDP+VNB	0.80 (0.66-0.96)	0.017	8.6
CALGB9633^[10]^	344	Ⅲ	Ⅰb	CBDCA+PTX	0.83 (0.64-1.08)^*^	NS	2
LACE^[11]^	4, 584	*Meta*-analysis	Ⅰ-Ⅲ	CDDP based	0.89 (0.82-0.96)	0.005	5.8
WJSG(Ⅱ)^[15]^	323	Ⅲ	Ⅰ-Ⅲ	UFT	0.55 (0.36-0.86)	0.022	15.1
JLCRG^[16]^	979	Ⅲ	Ⅰ (Ad)	UFT	0.71 (0.52-0.98)	0.04	3
NSCLC: non-small cell lung cancer; CI: confidence interval; CDDP: Cisplatin; NS: not significant; ALPI: Adjuvant Lung Project Italy; MVP: mitomycin+vindesin+cisplatin; BLT: Big Lung Trial; IALT: International Adjuvant Lung Cancer Collaborative Group Trial; Vin: vinca alkaloid; VNB: vinorelbine; ANITA: Adjuvant Navelbine International Trialist Association; CALGB: Cancer and Leukemia Group B; CBDCA: carboplatin; PTX: paclitaxel; LACE: Lung Adjuvant Cisplatin Evaluation; WJSG: West Japan Study Group for Lung Cancer Surgery; uft: uracil-tegafur; JLCRG: Japan Lung Cancer Research Group; Ad: adenocarcinoma. ^*^: 90%CI.							

**2:** Ongoing clinical trials with individualized adjuvant chemotherapy of NSCLC

Clinical trial	Stage	Prospectively tested marker	Trial description
TASTE	Ⅰ-Ⅲa	ERCC1 expression by IHC, *EGFR* mutations	Phase Ⅱ CDDP/ERL
SWOG 0720	Ⅰ	ERCC1 and RRM1 expression by automated AQUA immunofluorescence score	Phase Ⅱ Follow up or ACT according to risk profile
ITACA	Ⅱ-Ⅲa	ERCC1 and thymidylate synthase expression by qRT-PCR	Phase Ⅲ ACT assigned according to markers expression
SCAT	Ⅰ-Ⅲa	BRCA1 expression by qRT-PCR	Phase Ⅲ CDDP DOC *vs* “gene assigned CT”
qRT-PCR: quantitative reverse transcription-polymerase chain reaction; IHC: immunohistochemistry; ACT: adjuvant chemotherapy; CDDP: cisplatin; DOC: docetaxel; ERCC: excision repair cross-complementation gene; RRM: ribonucleotide reductase M1 gene; AQUA : *in situ* protein expression using accurate quantitative analysis.			
